# Updates in Diagnostic Tools for ILD

**DOI:** 10.3390/jcm14092924

**Published:** 2025-04-24

**Authors:** Arsal Tharwani, Manuel L. Ribeiro Neto

**Affiliations:** Department of Pulmonary Medicine, Integrated Hospital Care Institute, Cleveland Clinic, Cleveland, OH 44195, USA; tharwaa@ccf.org

**Keywords:** interstitial lung diseases, biopsy, diagnosis

## Abstract

Interstitial lung disease (ILD) is a group of diffuse parenchymal disorders, which are diagnosed in many cases by multidisciplinary discussion (MDD). In some cases, diagnosis can be challenging, and the addition of histopathology can increase diagnostic confidence. The tools to obtain a histopathological sample to diagnose ILD are expanding. In this review, we will discuss the various modalities, their sensitivities and specificities, and procedural complication rates. In this review, we conducted a comprehensive review of literature focusing on emerging and established diagnostic tools for ILD. A systematic search of peer-reviewed publications was performed using PubMed with a focus on clinical trials, retrospective and prospective cohort studies, and systematic reviews. The key diagnostic modalities in focus were genomic classifier (GC), transbronchial cryobiopsy (TBLC), surgical lung biopsy (SLB), endobronchial ultrasound cryobiopsy (EBUS-C), genetic testing, and speckled transthoracic echocardiography (STE). Data extracted from these studies focused on diagnostic yield, specificity, sensitivity, and procedural complication rate. Genomic classifier, a gene-based molecular diagnostic tool, has a high specificity for histological usual interstitial pneumonia (UIP). However, in cases of a negative result, it often results in a need for further invasive sampling by TBLC or SLB. TBLC results in a larger histological sample, which can increase diagnostic yield and increase diagnostic confidence at MDD. Recent prospective trials have compared this modality with SLB and found 63–77% interobserver agreement between pathologists. SLB remains the gold standard with diagnostic yields reported to be more than 90%. EBUS-C has shown promising results increasing diagnostic yield in patients with suspected sarcoidosis or lymphoma. All diagnostic modalities have procedural complications with most common being pneumothorax, bleeding and, rarely, death. Advancements in diagnostic tools for interstitial lung disease (ILD) have significantly improved accuracy. Even though surgical lung biopsy remains the gold standard, the alternative modalities are promising and provide a promising yield with a lower procedural risk.

## 1. Introduction

Interstitial lung disease (ILD) is a heterogenous group of diffuse parenchymal lung disorders with varying treatment responses and survival rates [1]. In most cases, the diagnosis is idiopathic pulmonary fibrosis (IPF), for which the mainstay of treatment is anti-fibrotic therapy [1,2,3]. Immunosuppression in IPF can lead to harm [4]. In other cases, such as hypersensitivity pneumonitis (HP) and sarcoidosis, removal of the offending agent (in HP) and immunosuppression are helpful [5,6]. Similarly, in connective tissue disease-associated ILD (CTD-ILD), the primary treatment is immunosuppression with or without anti-fibrotic therapy [2,3,7,8]. Therefore, an accurate diagnosis is key because it can have treatment and prognostic implications.

Most cases of ILD can be diagnosed with high confidence by a multidisciplinary discussion (MDD), involving clinical and radiological data [1,9]. When discussing a case with other pulmonologists and thoracic radiologists at the MDD before a lung biopsy is performed, for example, a consensus of a probable usual interstitial pneumonia (UIP) pattern and IPF could be reached with high confidence, avoiding unnecessary procedures [10]. However, in 10–40% of newly diagnosed ILD, the addition of histopathology, by a lung biopsy, may be needed to establish a diagnosis and/or improve diagnostic confidence [9]. In those cases, the MDD could assist in choosing the best diagnostic modality. For example, if non-specific interstitial pneumonia (NSIP) is in the differential, the MDD may recommend a surgical lung biopsy (SLB) over a transbronchial lung cryobiopsy (TBLC).

**Figure 1:**
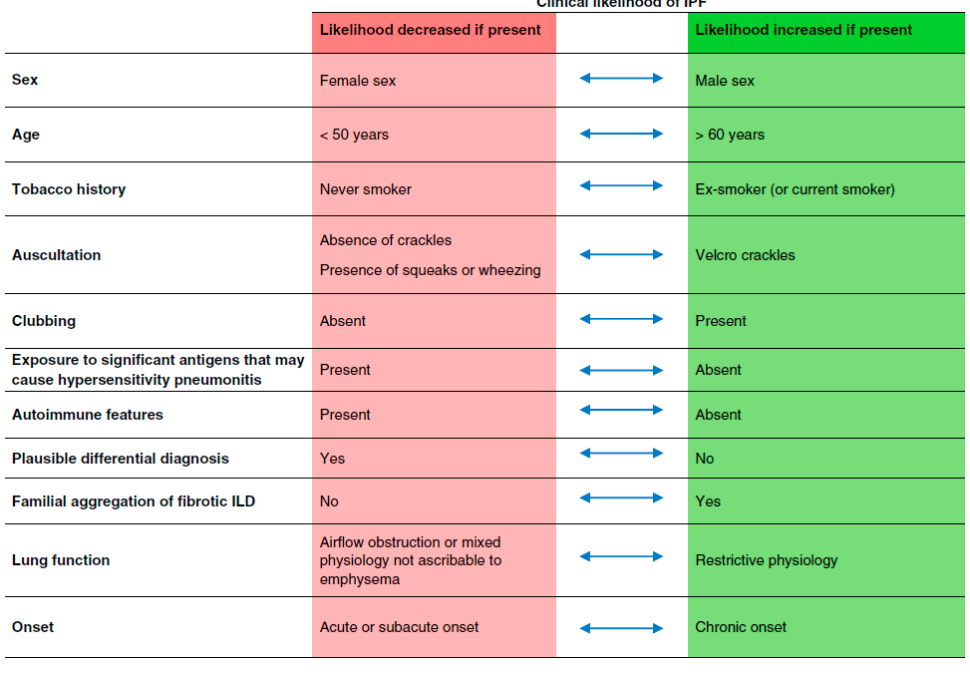
Clinical likelihood of IPF based on demographics and clinical features [11]. Legend. IPF, idiopathic pulmonary fibrosis; ILD, interstitial lung disease. Reprinted with permission of the American Thoracic Society, Copyright C 2025 American Thoracic Society. All rights reserved.

In IPF, a clinical likelihood assessment can be made based on certain risk factors, such as older age (>60 years of age), male sex, current or former tobacco smoking history, or Velcro crackles on auscultation (Figure 1) [11]. If these risk factors are present, it increases the pre-test clinical probability of IPF. On the other hand, exposure to antigens implicated with HP, the presence of autoimmune features, and acute/sub-acute onset decreases the pre-test clinical probability of IPF [11]. Clinicians can use these data to classify patients into a high (70–100%), intermediate (30–70%), or low (0–30%) pre-test clinical probability of IPF [11]. However, these clinical probability models have to be used with caution, since some features could be present in many different ILDs. For example, an UIP pattern can be seen in CTD patients. Prospective studies are needed to validate these models.

**Figure 2:**
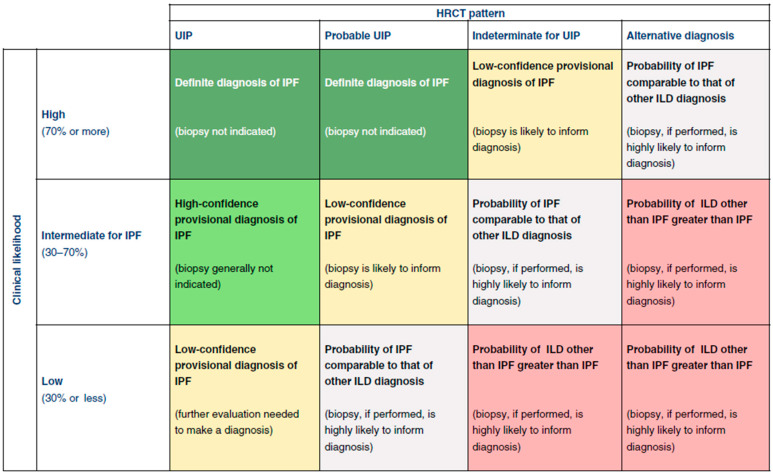
Probability of non-invasive diagnosis of IPF based on clinical diagnosis and radiographic data [11]. Legend. HRCT, high-resolution computed tomography; UIP, usual interstitial pneumonia; IPF, idiopathic pulmonary fibrosis. Reprinted with permission of the American Thoracic Society. Copyright C 2025 American Thoracic Society. All rights reserved.

The radiographic assessment of UIP in high-resolution computed tomography (HRCT) should classify patients into one of the four categories: UIP (presence of basilar and peripheral reticulations, honeycombing with or without traction bronchiectasis), probable UIP (presence of basilar and peripheral reticulations, traction bronchiectasis without honeycombing), indeterminate for UIP (diffuse fibrosis distribution that do not suggest any specific etiology), or HRCT suggesting an alternative diagnosis (with features including presence of ground glass changes, cysts, consolidations, nodules). In a high pre-test clinical probability of UIP, radiographic patterns consistent with UIP or probable UIP are suggestive of IPF without a need for biopsy (Figure 2). In an intermediate pre-test clinical probability of IPF, an HRCT pattern consistent with UIP can be used to confirm a diagnosis of IPF without a need for biopsy. In all other cases, a biopsy may be needed for better assessment.

The decision to pursue biopsy in these undifferentiated cases of ILD should be weighed against the patient’s risk of procedural complications and should be considered if the biopsy is going to result in a change in clinical management: for example, differentiating fibrotic-HP from IPF or CTD-ILD from IPF [12]. These forms of fibrotic ILDs can present with a progressive fibrosis phenotype, for which a biopsy is essential to exclude IPF from the other types (such as HP or CTD-ILD) because treatment is going to be vastly different [12,13], In other cases of undifferentiated ILD, atypical radiological patterns, such as extensive consolidation, ground glass changes, cysts, and nodules may require a histopathological assessment if no clear etiology has been identified on clinical or radiological evaluation [14]. Furthermore, the presence of histological UIP on histopathology has been shown to have a progressive phenotype and is likely to result in adverse outcomes [15]. Hence, in these scenarios, having histopathology can also help in the prognostication of ILD. In the past, SLB was the preferred method for histopathological diagnosis [16,17,18]. With the advent of newer and less invasive methods, such as genomic classifier (GC) and TBLC, pulmonologists have a wide range of newer tools available. Despite the availability of newer modalities, there is a lack of guidance on what modality to use for clinical situations. In this chapter, we will discuss the tools available for diagnosing ILD, their use in clinical practice, their sensitivity, specificity, and procedural complication rates. Table 1 shows a summary of the pros and cons of the modalities discussed herein.

## 2. Genomic Classifier

The reliable identification of UIP in conventional transbronchial forceps biopsies (TBB) has been challenging due to an inadequate sampling of alveoli and lung parenchyma [19]. In retrospective studies, the overall yield has been variable and reported to be less than 10% by some, while others have reported a yield of 30–43% [20,21,22]. Given the poor yield of conventional TBB, Parkantz et al. described the addition of a machine-learning methodology with TBB to train an algorithm to differentiate UIP from non-UIP [23]. This was the Envisia Genomic Classifier (Veracyte, San Francisco, CA, USA), which is a gene expression based molecular diagnostic tool, which utilizes RNA sequencing to detect molecular UIP gene expression signature [23,24]. The output is a binary result, UIP or non-UIP.

The initial study by Parkantz et al. described a test specificity of 86% and sensitivity of 63% [23]. In a prospective study by Raghu et al., the classifier identified UIP in TBB samples in 49 patients with 88% specificity and 70% sensitivity [24]. Among these patients, 42 had possible or indeterminate UIP patterns on HRCT. The GC also increased diagnostic confidence, especially in cases where the histopathology was non-diagnostic [24]. Similarly, Richeldi et al. showed a sensitivity and specificity of 60% and 92%, respectively [25]. When used in conjunction with HRCT patterns of UIP, the sensitivity of the test improved to 79% [25]. Pooling data from all prior studies on genomic classifier, a meta-analysis reported a combined sensitivity of 68% and specificity of 92% [26].

Given the high specificity of the test, the results can be useful in cases of probable and/or indeterminate UIP pattern by HRCT, where the diagnostic confidence of IPF is low in MDD settings. In a study, the addition of GC increased diagnostic confidence for IPF from 30% to 69% [27,28]. The concurrent increase in IPF diagnosis led to an increase in recommendation of antifibrotic from 10% to 46% in this cohort [27,28]. Therefore, it reduces the need for further invasive sampling by TBLC or SLB. The use of a genomic classifier can also enhance understanding regarding prognostication and disease progression for ILD, as shown by Chaudhry et al. [13]. A positive GC result of UIP was significantly associated with a higher risk of progression and annual FVC decline [13]. This finding may influence the decision to pursue antifibrotics as a management strategy. Hence, the role of GC is expandable beyond diagnostic data and applicable to management as well. Furthermore, it can also be utilized in limited resource centers without access to ILD experts.

However, the test has a low sensitivity, and false negatives are common. Hence, in cases where the GC results are a non-UIP result, it will likely result in a need for a more invasive sampling by TBLC or SLB.

The complication rate from GC is related to the risk of conventional TBB. The main complications are bleeding and pneumothorax. In general, the risk of pneumothorax is 5% [12,29]. However, in pulmonary fibrosis with resting hypoxia and severe reduction in DLCO, the risk can be increased to 7% [30]. The risk of major bleeding requiring a balloon placement, causing hemodynamic or respiratory disturbance, or termination of the procedure, is between 1 and 3% [29,31]. The rates of these complications are still considerably lower than those of TBLC and SLB.

In summary, the GC has a high specificity for histological UIP, which has a high predictive value for IPF. However, caution must be exercised in interpretation of a GC positive UIP result because non-IPF ILDs, such as fibrotic HP and CTD-ILD, can have a histological UIP pattern. Most importantly, a GC negative UIP result does not rule out UIP due to its low sensitivity and high chance of a false negative test result.

## 3. Transbronchial Cryobiopsy

Transbronchial cryobiopsy has been increasingly utilized as an alternative diagnostic technique across many centers. Cryoprobes utilize cooling agents (e.g., carbon dioxide or nitric oxide) under high pressure, which leads to gaseous expansion and to the pulmonary tissue adhering to the cold probe tip. This results in a larger sample available for histopathological analysis than the one obtained through conventional TBB. The initial meta-analysis reported a diagnostic yield of TBLC ranging from 70% to 84% as compared to SLB’s yield of >90% [32,33,34]. However, these studies were mainly retrospective, and some were prospective cohort studies without a direct comparison to SLB. Romagnoli et al. attempted a direct comparison study between TBLC and SLB in the CryoPID study and showed a poor interobserver agreement of 38% (kappa 0.22) [35]. However, this study was underpowered with a small sample size (N = 21) and utilized only one pathologist for histopathology review.

The COLDICE study was a prospective cohort study with sequential TBLC and SLB for histopathological and MDD agreement [36]. The pathologists were blinded to the clinical and radiographic data, which was followed by discussion at MDD regarding the final diagnosis. Histopathological agreement was 71% (kappa 0.7) and diagnostic agreement at MDD was 77% (kappa 0.62) with a high degree of concordance [36]. For TBLC with high or definitive confidence at MDD, there was a 95% concordance with SLB [36]. In cases with low confidence or non-diagnostic TBLC, SLB helped inform decisions in 23% of cases [36]. The predominant diagnosis at MDD was IPF, which was followed by HP [36].

The COLD study was the first randomized-controlled trial comparing TBLC and SLB. It compared a step-up strategy, TBLC followed by SLB, with an immediate SLB for diagnosis of ILD after MDD discussion [37]. The diagnostic yield of TBLC was 82% [37]. TBLC was inconclusive in 11% (3/28) of cases, necessitating SLB for diagnosis [37]. The overall histopathological agreement between pathologists for the lung biopsies was 63% (kappa 0.53), being 61% in the step-up strategy (kappa 0·53) and 65% in the immediate SLB group (kappa 0.51) [37]. Predominant diagnoses were HP and idiopathic non-specific interstitial pneumonia (NSIP) [37].

The CAN-ICE study looked at the interobserver agreement between TBLC and SLB within and between academic centers in The Netherlands [38]. They showed a moderate agreement between TBLC-MDD and SLB-MDD with 62% of concordance (kappa 0.46). Discordance in MDD was related to the TBLC diagnosis of IPF, which was classified as HP on SLB. TBLC was also likely to yield more unclassifiable ILD. Between-center agreement was better for SLB (kappa 0.71) than TBLC (kappa 0.29). 

The use of TBLC and GC has been investigated by a prospective study, which showed that the addition of GC to TBLC increased diagnostic confidence in MDD for probable UIP pattern and diagnosis of IPF [39]. The diagnostic confidence of IPF increased from 31% to 92% after the addition of GC to TBLC [39]. However, for indeterminate patterns of UIP, the addition of GC to TBLC made no significant difference in diagnostic confidence. In these cases, TBLC by itself helped increase diagnostic confidence in MDD [39]. Hence, in cases with a radiographic patten of probable UIP, the addition of GC to TBLC can help increase diagnostic confidence, especially in cases of non-diagnostic histopathology.

The decision to pursue a TBLC depends on a variety of factors. First, is the institution comfortable with the procedure both from procedural (given potential complications) and pathology expertise (due to its lower sample size compared to SLB) standpoints? Second, can it be determined based on the differential diagnoses being considered, such as NSIP, HP, cystic lung diseases or an indeterminate pattern for UIP [14]? Third, patients who are likely to be poor SLB candidates (due to respiratory disease or comorbidities) can benefit from TBLC [14]. The European respiratory society recommends TBLC as the first step for obtaining a histopathological sample in undifferentiated ILD [14].

Major complications associated with TBLC are major airway bleeding and pneumothorax. Based on pooled data from meta-analyses, the risk of pneumothorax from the procedure requiring a chest tube is 5.6% [10,36,37,38,40]. In cases requiring chest tube drainage for pneumothorax, the chest tube is usually removed within 24 h. Compared to SLB, the duration of chest tube placement is shorter, the hospital stay is shorter, and the infection risk is lower in TBLC [10,37,38].

Unfortunately, due to the lack of standardization of grades of airway bleeding during and post-procedure, the complication rates are variable for airway bleeding. Moderate bleeding requiring bronchoscopic intervention with the instillation of cold saline and/or reinflation of balloon is reported to be between 22 and 36% [36,37,38]. Severe airway bleeding requiring reintubation, bronchial blocker, angioembolization or procedural abortion was a rare event in these studies and has been reported to be 1.6% based on meta-analyses [36,37,38,40]. However, in one study, severe airway bleeding requiring reintubation and bronchial blocker placement was reported to be around 12% [10]. Mortality from TBLC has been estimated to be 0.4% [39].

## 4. Surgical Lung Biopsy

Surgical lung biopsy remains the reference standard for the diagnosis of ILD. The most common technique is video-assisted thoracoscopy (VATS) with diagnostic yields reported to be around 95% [41]. However, the decision to pursue a VATS for a diagnosis is a complicated one, considering the morbidity and mortality of the procedure.

In-hospital mortality after SLB has been reported to be 1.7% for elective procedures across 10-year studies (from 1990s to 2000s) in the United States and United Kingdom [16,42]. The 30-day and 90-day mortality for the procedure can approach 2.4% and 3.9%, respectively [16,43]. For non-elective procedures, the in-hospital mortality rates are considerably higher with estimates of around 16% [42]. Certain patient factors, such as advanced age, higher Charleston comorbidity scores and pre-operative respiratory failure, have been associated with increased risk of mortality after VATS [16,42,43,44].

A recent study from the United States looked at cases between 2012 and 2019, and it did not report any mortality for elective procedures after VATS [45]. For patients who were hospitalized, post-operative mortality was 10% [45]. Similarly, a prospective study from France did not report any post-operative mortality for patients undergoing elective VATS [18]. The lower rates of mortality, as compared to earlier studies, can be attributed toward refined surgical techniques and patient selection criteria [18].

The clinical decision to pursue an SLB should be based on patient risk factors, such as age, comorbidities, the physiological severity of ILD, and baseline hypoxemia. It is also dependent on a clinical center’s diagnostic yield from TBLC. The decision to pursue an SLB may further be enforced if a TBLC is inconclusive and the addition of histopathology to a case will influence management decisions. Morbidity after VATS is common and has been reported as bleeding, pneumothorax, infection and prolonged hospitalization [17,42,45,46]. The rate of recurrent or persistent pneumothorax is 5.5% [40]. The risk of empyema or pneumothorax is 2.1% [40]. The risk of 30-day mortality is 1.7% [40]. Morbidity is lower for elective procedures as opposed to the hospitalized cohort. However, morbidity rates, such as acute exacerbation of ILD (6.1%), persistent air leak (5.9%), respiratory infection (6.5%), and delayed wound healing (3.3%) are higher than those for transbronchial cryobiopsy [12,47].

In summary, SLB remains the reference standard for ILD diagnosis. Given the relatively high risk of complications from the procedure, a thorough review of patient factors, such as age, comorbidities, along with the nature of the procedure (elective vs. emergent) and use of an MDD discussion, should be considered.

## 5. Genetic Testing

Genetic predisposition to pulmonary fibrosis has been confirmed by the discovery of several key gene mutations [48,49,50]. Monogenic mutations, such as genes involved in telomere or surfactant hemostasis, namely telomere-related genes (TRGs) or surfactant-related genes (SRGs), have been identified in familial pulmonary fibrosis (FPF) [48,50].

Mutations in TRG are associated with pathological chromosomal shortening, which results in pulmonary and extra-pulmonary manifestations. The prototypical manifestation of a homozygous TRG mutation is dyskeratosis congenita, which is associated with abnormal skin pigmentation, oral leukoplakia, nail dystrophy and bone marrow failure [51]. In heterozygous TRG mutations, predominantly found in adults, extra-pulmonary abnormalities involving the hematological and hepatic systems are common [48,49,50]. It can also be associated with the premature graying of hair. Pulmonary fibrosis subtypes in TRG mutations include IPF, NSIP, fibrotic HP, and CTD-ILD [52]. Mutations in TRG are associated with poor outcomes in IPF and in lung transplantation. Apart from prognosis, treatment with immunosuppression for pulmonary fibrosis in TRG is associated with adverse outcomes [49].

Mutations in SRG are associated with various radiographic and histological variants, namely UIP, NSIP and desquamative interstitial pneumonia [50]. Since surfactant production is limited to the lungs, these mutations do not cause extra-pulmonary manifestations; however, they are associated with increased risk of lung cancer, especially adenocarcinoma [53].

Genomics-based testing options include genetic testing and telomere length measurement. Testing for the telomere length of peripheral leukocytes and reporting as age-adjusted values is one of the tools available [49]. Mutations in TRG may present with short telomere length, which is usually less than that in the 25th percentile [54]. However, some mutation variants in TRG present with normal telomere length, which does not rule out the disorder [54]. In these cases, genomic sequencing, by whole genome, whole exon or panel sequencing, for previously recognized high-risk genes can give valuable information [50]. For SRG mutations, genomic sequencing is the only way to test [55].

Single nucleotide polymorphisms (SNPs) have also been identified in fibrotic ILD patients. The most studied is the MUC5B promoter gene rs35705950, which has been found in patients with IPF, FPF, rheumatoid arthritis (RA)-ILD, and fibrotic HP [48]. MUC5B promoter variant identified in IPF is associated with better survival and slower progression [56]. It is not associated with better survival in familial or early-onset fibrosis [49,56].

Unfortunately, there is no consensus on when to test individuals for TRG and SRG mutations. In patients with a clinical suspicion, the European Respiratory Society recommends testing if fibrosing ILD is detected before the age of 50, when a first- or second degree relative has fibrotic ILD, or in any patient with a relative carrying a known pathogenic variant [49]. Treatment of these pathogenic variants includes anti-fibrotic therapy and/or early referral for lung transplantation [50].

## 6. Endobronchial Ultrasound-Guided Cryobiopsy

In one specific ILD, sarcoidosis, a biopsy of thoracic lymph nodes may be enough to confirm its diagnosis. Endobronchial ultrasound-guided cryobiopsy (EBUS-C) is a recent innovation that can potentially increase the diagnostic yield of lymph node biopsies in suspected sarcoidosis patients.

This has been demonstrated by a retrospective Italian cohort that included 48 patients who had both EBUS-C and EBUS-transbronchial needle aspiration (TBNA) in the same procedure for mediastinal or hilar lesions. For sarcoidosis, the diagnostic yield of EBUS-C was higher than that of EBUS-TBNA (93% versus 79%, respectively) [57]. Those findings were corroborated by a larger prospective European cohort that included 137 patients with mediastinal or hilar lesions. In that study, the diagnostic yield for sarcoidosis of EBUS-C was also higher than that of EBUS-TBNA (93% versus 41%, respectively). Importantly, EBUS-C also had a higher diagnostic yield for “uncommon tumors” including lymphoma [58].

In a separate prospective Spanish cohort, however, different results were demonstrated. Fifty patients with mediastinal and hilar lesions were included in that study, and both EBUS-C and EBUS-TBNA identified all six patients with sarcoidosis. The overall diagnostic yield, however, was higher for EBUS-C compared to EBUS-TBNA (96% versus 82%, respectively), which was driven by lymphoma and other malignancies [59].

When applying these data to patient care, we also suggest that clinicians consider the pre-test probability of sarcoidosis before the bronchoscopy. If the pre-test probability is high, with a typical pattern of thoracic adenopathy, EBUS-TBNA is probably enough to achieve an accurate diagnosis of sarcoidosis. In atypical cases, especially when lymphoma is being suspected, EBUS-C is a reasonable option and should be considered.

## 7. Speckled Transthoracic Echocardiography

The right ventricle (RV) can face an increase in afterload in patients with fibrotic ILD due to hypoxemic vasoconstriction and architectural destruction, which can lead to reduction in RV function and lead to RV–arterial uncoupling [60]. The use of speckle tracking echocardiography (STE) to analyze global myocardial strain has been studied to prognosticate patients with fibrotic ILD [61,62,63].

Using parameters, such as RV global longitudinal strain (RV GLS) and lateral wall longitudinal strain (LWLS), earlier signs of RV dysfunction can be detected in patients with early IPF [62]. These parameters are available using speckle tracking echocardiography (STE). The change in these parameters becomes pronounced on exercise in patients with IPF as compared to patients without fibrotic interstitial lung disease [62]. Parameters of standard echocardiography, such as tricuspid annular plane systolic excursion (TAPSE) and RV tissue doppler velocity (TD), are not sensitive enough to detect signs of early RV dysfunction [62].

Another study used RV function metrics, such as the ratio of tricuspid annular plane systolic excursion (TAPSE) or RV global longitudinal strain (GLS) to the systolic pulmonary arterial pressure (sPAP), to assess RV–arterial coupling. The study found that the RV GLS/sPAP is a reliable non-invasive parameter of RV–arterial uncoupling and is associated with worse outcomes in patients with fibrotic ILD [61]. Among patients with fibrotic ILD with RV–arterial uncoupling, patients with IPF tend to have the poorest prognosis. Among patients with IPF, left ventricular (LV) diastolic dysfunction was common, which was commonly assessed by using LV global longitudinal strain [63].

Hence, STE has a role in patients with fibrotic ILD, particularly IPF, in looking for signs of RV–arterial uncoupling, which can help prognosticate these patients for the clinicians.

## 8. Putting Everything Together

**Figure 3:**
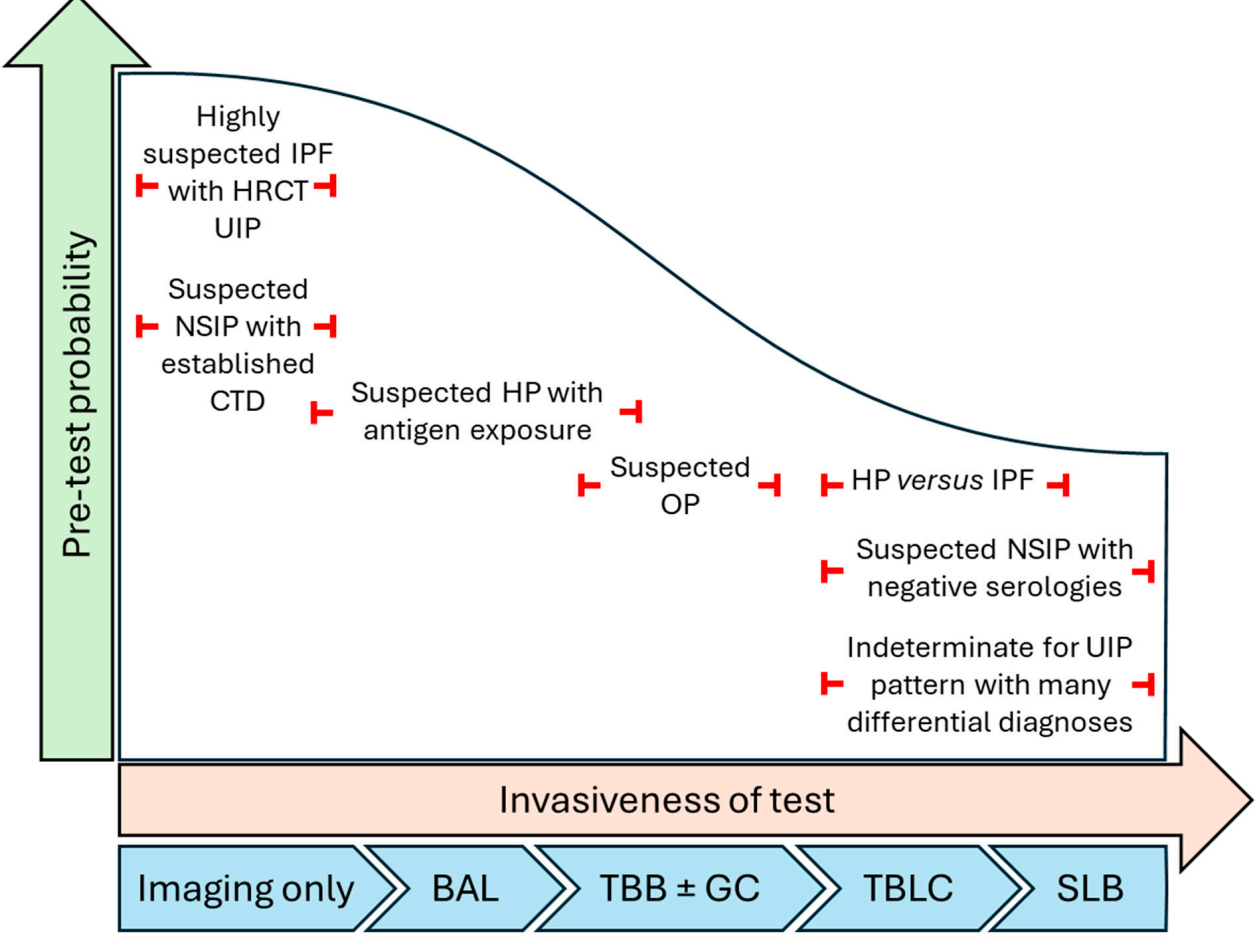
Relationship between pre-test probability and invasiveness of tests. Legend. NSIP, non-specific interstitial pneumonia; HP, hypersensitivity pneumonitis; IPF, idiopathic pulmonary fibrosis; UIP, usual interstitial pneumonia; OP, organizing pneumonia; HRCT, high-resolution computed tomography; CTD, connective tissue disease; SLB, surgical lung biopsy; TBLC, transbronchial lung cryobiopsy; TBB, transbronchial biopsy; GC, genomic classifier; BAL, bronchoalveolar lavage.

With so many tools available to diagnose ILD, the decision-making process may be overwhelming for both clinicians and patients. A simple approach is to decide how high the pre-test probability of the leading diagnosis is, and what are the differential diagnoses in each case. Depending on those two variables, the diagnostic pathway will lead clinicians and patients to different decisions. For example, in a patient with a high pre-test probability of IPF (no clinical features of alternative diseases) and a UIP pattern on HRCT, no biopsy is needed. In a patient with highly suspected fibrotic HP and an obvious antigen exposure, either imaging alone or a bronchoalveolar lavage with lymphocytosis may be enough to establish a confident diagnosis. In a patient with suspected organizing pneumonia, a conventional forceps TBB may easily yield the correct diagnosis. The fact that we can use all of those tools does not mean that we have to. However, if the pre-test probably is not high enough for any disease, and the differential diagnoses are many, the use of the diagnostic tools discussed above are certainly welcome (Figure 3).

Another important aspect is to consider patient’s values and preferences during this process. If a patient’s goal is to achieve an accurate diagnosis as fast as possible, with a single procedure, and he or she is willing to accept the risks, an SLB may be the best option for that patient in most situations. On the other hand, if a patient’s goal is to avoid procedural risks, and he or she is willing to undergo two separate procedures if needed, a TBLC may be the best option for that patient before an SLB.

In this review, we have a generalized assumption that GC, TBLC and SLB expertise is available to the clinical centers. These procedures are governed by the technique of the bronchoscopist and thoracic surgeon, which influences diagnostic yield. Hence, this is subject to bias and may introduce variability in diagnostic results.

Finally, we must keep in mind that some degree of uncertainty may always exist in some ILD cases, as it is inherently part of the process. Neither is the interobserver agreement between thoracic radiologists perfect, nor is the interobserver agreement between pathologists perfect as shown above. Therefore, ILD patients and caregivers need to be comfortable with the imperfect characteristics of these tests.

## 9. Future Directions

There are multiple tools available for the diagnosis of ILD. Unfortunately, given the large number of different ILDs and the recency of some of the diagnostic tools, there is no clear or simple algorithm for which technique to perform in every diagnosis. Even though the histological yield is higher with surgical lung biopsy, the availability of alternative tools is promising. Further research is needed to continue to improve the way patients with ILD are diagnosed. For example, more research is needed clarifying the use of TBLC in NSIP or to differentiate fibrotic HP versus UIP. Additional studies are also needed to determine the clinical utility of the GC, and more studies are needed to help clinicians decide which suspected sarcoidosis patients need an EBUS-C versus an EBUS-TBNA. As those and other questions are answered with future studies, we will be able to better use these important diagnostic tools.

## Figures and Tables

**Table 1:** Pros and cons of diagnostic tools.

Diagnostic Tool	Pros	Cons
GC	Good specificity for UIP (around 90%) Lower risk compared to TBLC or SLB	Availability indefinitely on hold by manufacturer Poor sensitivity for UIP, so many false-negative tests It cannot differentiate different causes of UIP (e.g., IPF versus HP versus CTD-ILD)
TBLC	Good diagnostic yield (around 80%) Lower risk compared to SLB	Learning curve exists Higher risk compared to TBB/GC Conflicting data in its ability to differentiate UIP from fibrotic HP Conflicting data in its ability to diagnose NSIP Patient may still require an SLB if TBLC is nondiagnostic
SLB	Highest diagnostic yield (>90%)	Highest risk
Genetic testing	May help diagnose FPF Noninvasive test May help identify patients who should not be on immunosuppression	Variable coverage by insurance companies It cannot diagnose specific patterns, since both TRG and SRG mutations can cause different patterns
EBUS-C	Diagnostic yield for sarcoidosis probably higher than with EBUS-TBNA Diagnostic yield for lymphoma or uncommon tumors is higher than with EBUS-TBNA	Learning curve exists Increase in procedure time Futile in most patients with suspected sarcoidosis

Legend. GC, genomic classifier; TBLC, transbronchial lung cryobiopsy; SLB, surgical lung biopsy; EBUS-C, endobronchial ultrasound-guided cryobiopsy; UIP, usual interstitial pneumonia; FPF, familial pulmonary fibrosis; TBNA, transbronchial needle aspiration; IPF, idiopathic pulmonary fibrosis; HP, hypersensitivity pneumonitis; CTD-ILD, connective tissue disease-related interstitial lung disease; TBB, transbronchial biopsy; NSIP, non-specific interstitial pneumonia; TRG, telomere-related genes; SRG, surfactant-related genes.

## References

[1] Raghu G., Remy-Jardin M., Richeldi L., Thomson C.C., Antoniou K.M., Bissell B.D., Bouros D., Buendia-Roldan I., Caro F., Crestani B. (2022). Idiopathic Pulmonary Fibrosis (an Update) and Progressive Pulmonary Fibrosis in Adults: An Official ATS/ERS/JRS/ALAT Clinical Practice Guideline. Am. J. Respir. Crit. Care Med..

[2] Flaherty K.R., Wells A.U., Cottin V., Devaraj A., Walsh S.L.F., Inoue Y., Richeldi L., Kolb M., Tetzlaff K., Stowasser S. (2019). Nintedanib in Progressive Fibrosing Interstitial Lung Diseases. N. Engl. J. Med..

[3] Maher T.M., Corte T.J., Fischer A., Kreuter M., Lederer D.J., Molina-Molina M., Axmann J., Kirchgaessler K.U., Samara K., Gilberg F. (2020). Pirfenidone in patients with unclassifiable progressive fibrosing interstitial lung disease: A double-blind, randomised, placebo-controlled, phase 2 trial. Lancet Respir. Med..

[4] Raghu G., Anstrom K.J., King T.E., Lasky J.A., Martinez F.J., Idiopathic Pulmonary Fibrosis Clinical Research Network (2012). Prednisone, Azathioprine, and N-Acetylcysteine for Pulmonary Fibrosis. N. Engl. J. Med..

[5] Spagnolo P., Rossi G., Trisolini R., Sverzellati N., Baughman R.P., Wells A.U. (2018). Pulmonary sarcoidosis. Lancet Respir. Med..

[6] Salisbury M.L., Myers J.L., Belloli E.A., Kazerooni E.A., Martinez F.J., Flaherty K.R. (2017). Diagnosis and treatment of fibrotic hypersensitivity pneumonia: Where we stand and where we need to go. Am. J. Respir. Crit. Care Med..

[7] Distler O., Highland K.B., Gahlemann M., Azuma A., Fischer A., Mayes M.D., Raghu G., Sauter W., Girard M., Alves M. (2019). Nintedanib for Systemic Sclerosis–Associated Interstitial Lung Disease. N. Engl. J. Med..

[8] Mathai S.C., Danoff S.K. (2016). Management of interstitial lung disease associated with connective tissue disease. BMJ.

[9] Tomassetti S., Ravaglia C., Puglisi S., Ryu J.H., Colby T.V., Cavazza A., Wells A.U., Pavone M., Vancheri C., Lavorini F. (2022). Impact of Lung Biopsy Information on Treatment Strategy of Patients with Interstitial Lung Diseases. Ann. Am. Thorac. Soc..

[10] Ribeiro Neto M.L., Arrossi A.V., Yadav R., Culver D.A., Mukhopadhyay S., Parambil J.G., Southern B.D., Tolle L., Pande A., Almeida F.A. (2022). Prospective cohort of cryobiopsy in interstitial lung diseases: A single center experience. BMC Pulm. Med..

[11] Cottin V., Tomassetti S., Valenzuela C., Walsh S.L.F., Antoniou K.M., Bonella F., Brown K.K., Collard H.R., Corte T.J., Flaherty K.R. (2022). Integrating Clinical Probability into the Diagnostic Approach to Idiopathic Pulmonary Fibrosis An International Working Group Perspective. Am. J. Respir. Crit. Care Med..

[12] Hariri L.P., Roden A.C., Chung J.H., Danoff S.K., Manjarres D.C.G., Hartwig M., Kheir F., King C., Kreider M., Lynch D.A. (2021). The Role of Surgical Lung Biopsy in the Diagnosis of Perspective from the Pulmonary Fibrosis Foundation. Ann. Am. Thorac. Soc..

[13] Ravaglia C., Nicholson A.G. (2021). Biopsy in interstitial lung disease: Specific diagnosis and the identification of the progressive fibrotic phenotype. Curr. Opin. Pulm. Med..

[14] Korevaar D.A., Colella S., Fally M., Camuset J., Colby T.V., Hagmeyer L., Hetzel J., Maldonado F., Morais A., Ravaglia C. (2022). European Respiratory Society guidelines on transbronchial lung cryobiopsy in the diagnosis of interstitial lung diseases. Eur. Respir. J..

[15] Goobie G.C., Guler S.A. (2023). The alternative approach: Genomic classifiers for prognostication in interstitial lung disease. Eur. Respir. J..

[16] Hutchinson J.P., McKeever T.M., Fogarty A.W., Navaratnam V., Hubbard R.B. (2016). Surgical lung biopsy for the diagnosis of interstitial lung disease in England: 1997–2008. Eur. Respir. J..

[17] Fisher J.H., Shapera S., To T., Marras T.K., Gershon A., Dell S. (2019). Procedure volume and mortality after surgical lung biopsy in interstitial lung disease. Eur. Respir. J..

[18] Radu D., Freynet O., Kambouchner M., Boubaya M., Nunes H., Uzunhan Y., Brillet P.Y., Guiraudet P., Noorah M.Z., Israël-Biet D. (2022). Diagnosis Yield and Safety of Surgical Biopsy in Interstitial Lung Diseases: A Prospective Study. Ann. Thorac. Surg..

[19] Katzenstein A.A., Mukhopadhyay S., Myers J.L. (2008). Erratum to “Diagnosis of usual interstitial pneumonia and distinction from other fibrosing interstitial lung diseases” [Hum Pathol 39 (2008) 1275-1294]. Hum. Pathol..

[20] Tomassetti S., Cavazza A., Colby T.V., Ryu J.H., Nanni O., Scarpi E., Tantalocco P., Buccioli M., Dubini A., Piciucchi S. (2012). Transbronchial biopsy is useful in predicting UIP pattern. Respir. Res..

[21] Shim H.S., Park M.S., Park I.K. (2010). Histopathologic findings of transbronchial biopsy in usual interstitial pneumonia.pdf. Pathol. Int..

[22] Berbescu E.A., Katzenstein A.-L.A., Snow J.L., Zisman D.A. (2006). Transbronchial Biopsy in Usual Interstitial Pneumonia. Chest.

[23] Pankratz D.G., Choi Y., Imtiaz U., Fedorowicz M., Anderson J.D., Colby T.V., Myers J.L., Lynch D.A., Brown K.K., Flaherty K.R. (2017). Usual Interstitial Pneumonia Can Be Detected in Transbronchial Biopsies Using Machine Learning. Ann. Am. Thorac. Soc..

[24] Raghu G., Flaherty K.R., Lederer D.J., Lynch D.A., Colby T.V., Myers J.L., Groshong S.D., Larsen B.T., Chung J.H., Steele M.P. (2024). Use of a molecular classifier to identify usual interstitial pneumonia in conventional transbronchial lung biopsy samples: A prospective validation study. Lancet Respir..

[25] Richeldi L., Scholand M.B., Lynch D.A., Colby T.V., Myers J.L., Groshong S.D., Chung J.H., Benzaquen S., Nathan S.D., Davis J.R. (2021). Utility of a Molecular Classifier as a Complement to High-Resolution Computed Tomography to Identify Usual Interstitial Pneumonia. Am. J. Respir. Crit. Care Med..

[26] Kheir F., Becerra J.P.U., Bissell B., Ghazipura M., Herman D., Hon S.M., Hossain T., Khor Y.H., Knight S.L., Kreuter M. (2022). Use of a Genomic Classifier in Patients with Interstitial Lung Disease A Systematic Review and Meta-Analysis. Ann. Am. Thorac. Soc..

[27] Lasky J.A., Case A., Unterman A., Kreuter M., Scholand M.B., Chaudhary S., Lofaro L.R., Johnson M., Huang J., Bhorade S.M. (2022). The Impact of the Envisia Genomic Classifier in the Diagnosis and Management of Patients with Idiopathic Pulmonary Fibrosis. Ann. Am. Thorac. Soc..

[28] Goobie G.C., Kass D.J. (2022). Genomic Classifiers in Diagnosing Interstitial Lung Disease: Finding the Right Place at the Right Time. Ann. Am. Thorac. Soc..

[29] Arya R., Boujaoude Z., Rafferty W.J., Abouzgheib W. (2020). Usefulness and safety of transbronchial biopsy with large forceps during flexible bronchoscopy. Baylor Univ. Med. Cent. Proc..

[30] Galli J.A., Panetta N.L., Gaeckle N., Martinez F.J., Moore B., Moore T., Courey A., Flaherty K., Criner G.J. (2017). Pneumothorax After Transbronchial Biopsy in Pulmonary Fibrosis: Lessons from the Multicenter COMET Trial. Lung.

[31] Pajares V., Núñez-Delgado M., Bonet G., Pérez-Pallarés J., Martínez R., Cubero N., Zabala T., Cordovilla R., Flandes J., Disdier C. (2020). Transbronchial biopsy results according to diffuse interstitial lung disease classification. Cryobiopsy versus forceps: MULTICRIO study. PLoS ONE.

[32] Sethi J., Ali M.S., Mohananey D., Nanchal R., Maldonado F., Musani A. (2019). Are Transbronchial Cryobiopsies Ready for Prime Time?. J. Bronchol. Interv. Pulmonol..

[33] Iftikhar I.H., Alghothani L., Sardi A., Berkowitz D., Musani A.I. (2017). Transbronchial Lung Cryobiopsy and Video-assisted Thoracoscopic Lung Biopsy in the Diagnosis of Diffuse Parenchymal Lung Disease A Meta-analysis of Diagnostic Test Accuracy. Ann. Am. Thorac. Soc..

[34] Johannson K.A., Marcoux V.S., Ronksley P.E., Ryerson C.J. (2016). Diagnostic Yield and Complications of Transbronchial Lung Cryobiopsy for Interstitial Lung Disease. Ann. Am. Thorac. Soc..

[35] Romagnoli M., Colby T.V., Berthet J., Gamez A.S., Mallet J., Serre I., Cancellieri A., Cavazza A., Solovei L., Amore A.D. (2019). Poor Concordance between Sequential Transbronchial Lung Cryobiopsy and Surgical Lung Biopsy in the Diagnosis of Diffuse Interstitial Lung Diseases. Am. J. Respir. Crit. Care Med..

[36] Troy L.K., Grainge C., Corte T.J., Williamson J.P., Vallely M.P., Cooper W.A., Mahar A., Myers J.L., Lai S., Mulyadi E. (2020). Diagnostic accuracy of transbronchial lung cryobiopsy for interstitial lung disease diagnosis (COLDICE): A prospective, comparative study. Lancet Respir. Med..

[37] Kalverda K.A., Ninaber M.K., Wijmans L., von der Thüsen J., Jonkers R.E., Daniels J.M., Miedema J.R., Dickhoff C., Hölters J., Heineman D. (2024). Transbronchial cryobiopsy followed by as-needed surgical lung biopsy versus immediate surgical lung biopsy for diagnosing interstitial lung disease (the COLD study): A randomised controlled trial. Lancet Respir. Med..

[38] Fortin M., Liberman M., Delage A., Dion G., Martel S., Rolland F., Soumagne T., Trahan S., Assayag D., Albert E. (2023). Transbronchial Lung Cryobiopsy and Surgical LungBiopsy: A Prospective Multi-Centre Agreement Clinical Trial(CAN-ICE). Am. J. Respir. Crit. Care Med..

[39] Kheir F., Alkhatib A., Berry G.J., Daroca P., Diethelm L., Rampolla R., Saito S., Smith D.L., Weill D., Bateman M. (2020). Using Bronchoscopic Lung Cryobiopsy and a Genomic Classifier in the Multidisciplinary Diagnosis of Diffuse Interstitial Lung Diseases. Chest.

[40] Rodrigues I., Gomes R.E., Coutinho L.M., Rego M.T., Machado F., Morais A., Bastos H.N. (2022). Diagnostic yield and safety of transbronchial lung cryobiopsy and surgical lung biopsy in interstitial lung diseases: A systematic review and meta-analysis. Eur. Respir. Rev..

[41] Han Q., Luo Q., Xie J., Wu L., Liao L. (2015). Diagnostic yield and postoperative mortality associated with surgical lung biopsy for evaluation of interstitial lung diseases: A systematic review and meta-analysis. J. Thorac. Cardiovasc. Surg..

[42] Hutchinson J.P., Fogarty A.W., Mckeever T.M., Hubbard R.B. (2016). In-Hospital Mortality after Surgical Lung Biopsy for Interstitial Lung Disease in the United States 2000 to 2011. Am. J. Respir. Crit. Care Med..

[43] Sigurdsson M.I., Isaksson H.J., Gudmundsson G., Gudbjartsson T. (2009). Diagnostic Surgical Lung Biopsies for Suspected Interstitial Lung Diseases: A Retrospective Study. Ann. Thorac. Surg..

[44] Carrillo G., Estrada A., Pedroza J., Aragón B., Mejía M., Navarro C., Selman M. (2005). Preoperative Risk Factors Associated With Mortality in Lung Biopsy Patients With Interstitial Lung Disease. J. Investig. Surg..

[45] Pastre J., Khandhar S., Barnett S., Ksovreli I., Mani H., Brown A.W., Shlobin O.A., Ahmad K., Khangoora V., Aryal S. (2021). Surgical Lung Biopsy for Interstitial Lung Disease. Ann. Am. Thorac. Soc..

[46] Hutchinson J., Hubbard R., Raghu G. (2019). Surgical lung biopsy for interstitial lung disease: When considered necessary, should these be done in larger and experienced centres only?. Eur. Respir. J..

[47] Ravaglia C., Bonifazi M., Wells A.U., Tomassetti S., Gurioli C., Piciucchi S., Dubini A., Tantalocco P., Sanna S., Negri E. (2016). Safety and Diagnostic Yield of Transbronchial Lung Cryobiopsy in Diffuse Parenchymal Lung Diseases: A Comparative Study versus Video-Assisted Thoracoscopic Lung Biopsy and a Systematic Review of the Literature. Respiration.

[48] Adegunsoye A., Kropski J.A., Behr J., Blackwell T.S., Corte T.J. (2024). Genetics and Genomics of Pulmonary Fibrosis Charting the Molecular Landscape and Shaping Precision Medicine. Am. J. Respir. Crit. Care Med..

[49] Al R.B.E.T., Borie R., Kannengiesser C., Antoniou K., Bonella F., Crestani B., Fabre A., Froidure A., Galvin L., Griese M. (2023). European Respiratory Society statement on familial pulmonary fibrosis. Eur. Respir. J..

[50] Zhang D., Newton C.A. (2021). Familial Pulmonary Fibrosis Genetic Features and Clinical Implications. Chest.

[51] Savage S.A., Alter B.P. (2009). Dyskeratosis Congenita. Hematol. Oncol. Clin. N. Am..

[52] Borie R., Kannengiesser C., De Fontbrune F.S., Gouya L., Nathan N., Crestani B. (2017). Management of suspected monogenic lung fibrosis in a specialised centre. Eur. Respir. Rev..

[53] Wang Y., Kuan P.J., Xing C., Cronkhite J.T., Torres F., Rosenblatt R.L., DiMaio J.M., Kinch L.N., Grishin N.V., Garcia C.K. (2009). Genetic Defects in Surfactant Protein A2 Are Associated with Pulmonary Fibrosis and Lung Cancer. Am. J. Hum. Genet..

[54] Alder J.K., Hanumanthu V.S., Strong M.A., DeZern A.E., Stanley S.E., Takemoto C.M., Danilova L., Applegate C.D., Bolton S.G., Mohr D.W. (2018). Diagnostic utility of telomere length testing in a hospital-based setting. Proc. Natl. Acad. Sci. USA.

[55] Van Moorsel C.H.M., Van Oosterhout M.F.M., Barlo N.P., De Jong P.A., Van Der Vis J.J., Ruven H.J.T., Van Es H.W., Van Den Bosch J.M., Grutters J.C. (2010). Surfactant Protein C Mutations Are the Basis ofa Significant Portion of Adult Familial PulmonaryFibrosis in a Dutch Cohort. Am. J. Respir. Crit. Care Med..

[56] Peljto A.L., Zhang Y., Fingerlin T.E., Shwu-Fan M., Garcia J.G.N., Richards T.J., Silveira L.J., Lindell K.O., Steele M.P., Loyd J.E. (2013). Association between the MUC5B promoter polymorphism and survival in patients with idiopathic pulmonary fibrosis. JAMA.

[57] Poletti V., Petrarulo S., Piciucchi S., Dubini A., De Grauw A.J., Sultani F., Martinello S., Gonunguntla H.K., Ravaglia C. (2024). EBUS-guided cryobiopsy in the diagnosis of thoracic disorders. Pulmonology.

[58] Mangold M.S., Franzen D.P., Hetzel J., Latshang T.D., Roeder M., Vesenbeckh S.M., Ulrich S., Gaisl T., Steinack C. (2024). Ultrasound-guided transbronchial cryobiopsy of mediastinal and hilar lesions: A multicenter pragmatic cohort study with real-world evidence. BMJ Open Respir. Res..

[59] Ariza-Prota M., Pérez-Pallarés J., Fernández-Fernández A., García-Alfonso L., Cascón J.A., Torres-Rivas H., Fernández-Fernández L., Sánchez I., Gil M., García-Clemente M. (2023). Endobronchial ultrasound-guided transbronchial mediastinal cryobiopsy in the diagnosis of mediastinal lesions: Safety, feasibility and diagnostic yield—Experience in 50 cases. ERJ Open Res..

[60] Chemla D., Castelain V., Hoette S., Creuzé N., Provencher S., Zhu K., Humbert M., Herve P. (2013). Strong linear relationship between heart rate and mean pulmonary artery pressure in exercising patients with severe precapillary pulmonary hypertension. Am. J. Physiol. Hear. Circ. Physiol..

[61] Santoro C., Buonauro A., Canora A., Rea G., Canonico M.E., Esposito R., Sanduzzi A., Esposito G., Bocchino M. (2022). Non-Invasive Assessment of Right Ventricle to Arterial Coupling for Prognosis Stratification of Fibrotic Interstitial Lung Diseases. J. Clin. Med..

[62] D’Andrea A., Stanziola A.A., Saggar R., Saggar R., Sperlongano S., Conte M., D’Alto M., Ferrara F., Gargani L., Lancellotti P. (2019). Right Ventricular Functional Reserve in Early-Stage Idiopathic Pulmonary Fibrosis: An Exercise Two-Dimensional Speckle Tracking Doppler Echocardiography Study. Chest.

[63] Buonauro A., Santoro C., Galderisi M., Canora A., Sorrentino R., Esposito R., Lembo M., Canonico M.E., Ilardi F., Fazio V. (2020). Impaired right and left ventricular longitudinal function in patients with fibrotic interstitial lung diseases. J. Clin. Med..

